# Occupational risk of SARS-CoV-2 infection among healthcare workers in Bangladesh: a multicenter hospital-based study and lessons for future epidemics

**DOI:** 10.1186/s41182-024-00663-8

**Published:** 2024-12-03

**Authors:** Ahamed Khairul Basher, Md Abdullah Al Jubayer Biswas, Aninda Rahman, Mahmudur Rahman, Fahmida Chowdhury, Md. Zakiul Hassan

**Affiliations:** 1 Infectious Diseases Division, International Centre for Diarrhoeal Disease Research, Bangladesh (Icddr,b), Dhaka, 1212 Bangladesh; 2 Global Health Development, EMPHNET, 69 Mohakhali, Dhaka, 1212 Bangladesh; 3 Communicable Disease Control, Ministry of Health and Family Welfare Government of Bangladesh, Dhaka, Bangladesh

**Keywords:** SARS-CoV-2, COVID-19, Healthcare workers, LMIC, Infectious disease, Risk, Pandemic, Bangladesh

## Abstract

**Background:**

Frontline healthcare workers (HCWs) were particularly vulnerable to contracting SARS-CoV-2 infection as a result of occupational exposure. There is a scarcity of data characterizing the risk of SARS-CoV-2 infection among HCWs, particularly in low-income hospital settings. This study aimed to assess the prevalence of COVID-19 among HCWs and identify associated risk factors.

**Methods:**

From July 2021 to July 2023, we enrolled HCWs from 13 primary, 2 secondary, and five tertiary care hospitals in four selected districts of Bangladesh. We collected information on demography and risk exposure in a face-to-face interview. We calculated the odds ratio to measure the risk using multivariable logistic regression.

**Results:**

We enrolled 3436 HCWs: 22% (747) physicians, 47% (1632) nurses, and 31% (1057) support staff. Most of the HCWs were female 67% (2292), and the mean age was 38.1, IQR = 29–44 years. Overall, 26% (889) of HCWs had lab-confirmed SARS-CoV-2 infection. Among HCWs, nurses accounted for the highest proportion of COVID-19 infections at 53% (473/1632). Physicians had a significantly higher risk of infection with an aOR of 3.08 (95% CI 2.42–3.93; *p* < .001) compared to support staff. HCWs who had direct exposure to COVID-19 patients were also at a higher risk, with a 1.93 times higher likelihood of infection ([aOR] = 1.93, 95% CI 1.50–2.47; *p* < .001), compared to HCWs who were not exposed.

**Conclusions:**

This study highlights the heightened vulnerability of HCWs to SARS-CoV-2 infection due to occupational exposure and indicates the risk of nosocomial transmission to patients and emphasizes the importance of implementing targeted infection control measures, such as improved workplace safety protocols and comprehensive training to tackle future pandemics of similar traits.

## Introduction

The COVID-19 pandemic has placed unprecedented pressure on healthcare systems worldwide, with healthcare workers (HCWs) being disproportionately affected [1–3]. According to the World Health Organization in 2021 (WHO), approximately 115,500 HCWs have died due to COVID-19-related complications globally [4]. The pandemic has exacerbated existing challenges within healthcare systems, particularly in low- and middle-income countries (LMICs), where the healthcare workforce is often under-resourced and overburdened [5].

In Bangladesh, the combination of a high population density and a critically low number of HCWs has significantly exacerbated the strain on the healthcare system during the COVID-19 pandemic. With over 161 million people densely packed into the country, particularly in urban areas, the rapid spread of the virus overwhelmed hospitals and healthcare facilities, which were already operating with a limited workforce [6]. Bangladesh has only nine physicians per 10,000 people, a figure that places it among the lowest in South Asia in terms of the physician–patient ratio [7]. On the other hand, according to WHO, since the first day of COVID-19 case detection in Bangladesh on 8 March 2020 to 27 November 2022 a total of 2,036,527 COVID-19 cases were confirmed by RT-PCR, GeneXpert, and Rapid Antigen tests, including 29,431 related deaths with a case fatality rate (CFR) of 1.45% [8]. As a result, this shortage of healthcare professionals, combined with the surge in COVID-19 cases, left hospitals overburdened and stretched beyond capacity. Not only scarce healthcare workforce make the situation dire but also because of a limited number of testing facilities, the testing rate (0.34%) was the second lowest in South Asia after Afghanistan [9]. The increased patient load and low detection of pathogens not only strained resources but also heightened the risk of infection among HCWs. The pressure to care for a large number of patients with limited support increased HCWs' exposure to the virus, making them more vulnerable to contracting COVID-19, particularly in environments where personal protective equipment (PPE) and infection control measures were inadequate. As a result, the already fragile healthcare system faced an additional burden, with a significant proportion of infections and deaths occurring among the healthcare workforce itself. In addition, HCWs reported experiencing higher workloads, psychological distress, social exclusion, and absence of coordination and management creating a double burden of the disease among this risk group [10].

Studies, primarily conducted in high-income countries, have indicated that HCWs employed in emergency departments, general medicine and surgery wards, infectious diseases departments, and other outpatient departments are at higher risk of contracting COVID-19 [11]. These settings often involve direct and prolonged contact with infected patients, increasing the likelihood of viral transmission. While rigorous infection prevention and control (IPC) measures, such as proper hand hygiene and the correct donning and doffing of personal protective equipment (PPE), can significantly reduce this risk, the effectiveness of these measures is contingent upon adequate knowledge, training, and resources [12]. Unfortunately, in many healthcare facilities, especially in resource-limited settings, the lack of sufficient PPE, gaps in IPC practices, and inadequate training can lead to cross-contamination, not only among HCWs but also between patients within the same facility [13].

While a substantial body of research has been published on the risks of COVID-19 infection among HCWs in high-income countries, there is a significant gap in knowledge regarding the situation in low- and middle-income countries (LMICs), including Bangladesh [14]. The challenges faced by HCWs in LMICs, where healthcare systems are often under-resourced and overburdened, differ markedly from those in more affluent nations. Despite the critical role that HCWs play in managing the pandemic, there is insufficient data on the prevalence of SARS-CoV-2 infections among this group in Bangladesh, as well as on the specific risk factors and the overall burden of disease within this context. The exact extent of this risk remains unclear, as do the contextual factors that may influence the effectiveness of interventions.

This study aims to address these gaps by estimating the prevalence of SARS-CoV-2 infection among HCWs in selected healthcare facilities in Bangladesh and identifying the associated risk factors. The prevalence will provide a snapshot of the existing extent of the infection and by understanding the risk of COVID-19 among the HCWs, will suggest a sustainable protective measure for workplace safety. In addition, by generating prospective data, the study seeks to provide a foundation for policy implementation regarding occupational safety and infection control. A deeper understanding of these issues, particularly within the context of LMICs like Bangladesh, is essential not only for managing the current pandemic but also for preparing for future epidemics. The findings of this study will inform targeted interventions that consider the unique challenges faced by healthcare systems in LMICs, thereby helping to prevent silent transmission of SARS-CoV-2 and other infectious diseases in healthcare settings.

## Methods

### Study design and participants

**Fig. 1:**
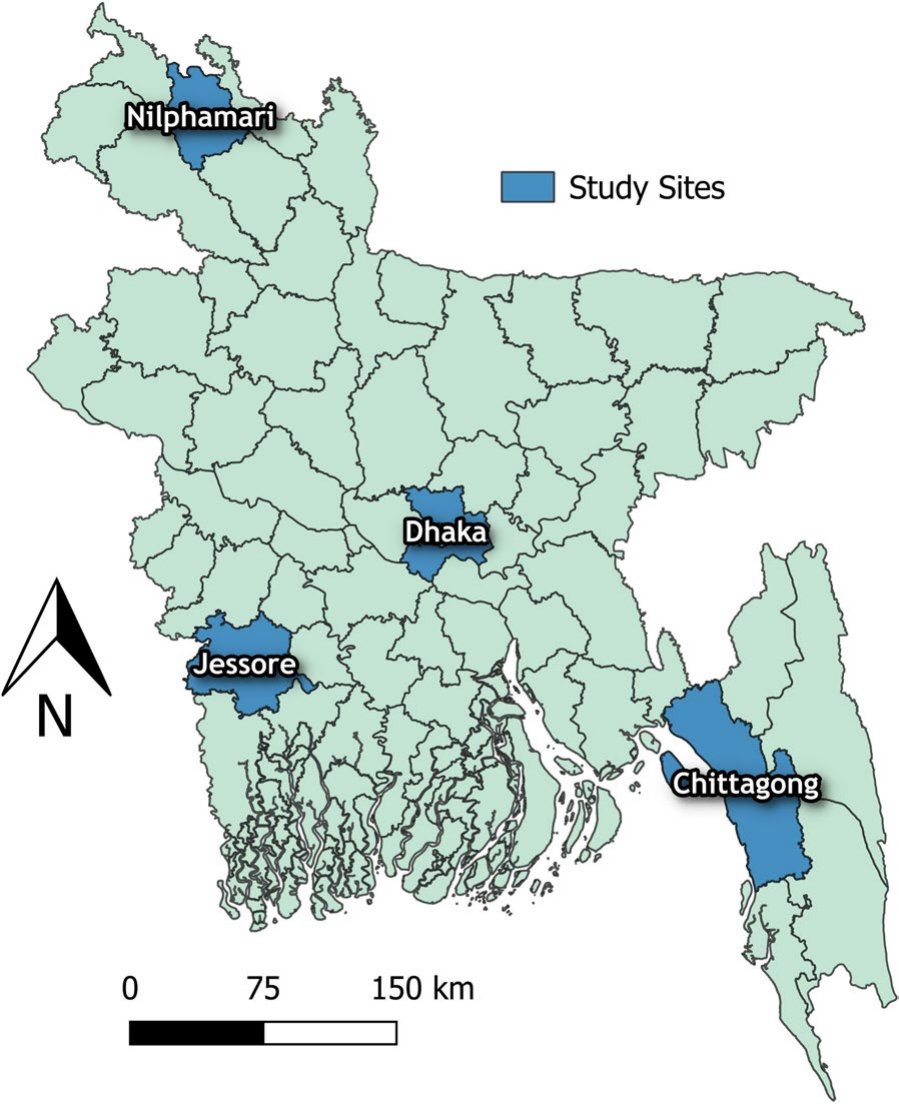
Spatial distribution of the study sites

We designed a cross-sectional study leveraging an ongoing cohort platform comprising HCWs of all designations (Physicians, Nurses, and support staff). From March 2021 to March 2023, we recruited HCWs from 20 healthcare facilities: primary 13, secondary 2, and tertiary 5. Among these 18 were public, and 2 were private (Fig. 1).

### Procedures

We approached all HCWs of study facilities who met the inclusion criteria and conducted a face-to-face interview session with a structured questionnaire who consented to participate voluntarily.

### Inclusion criteria

1. All the HCWs directly or indirectly involved in patient care.
2. All the participants who provide written informed consent.

### Exclusion criteria

1. All the administrative staff and staff from basic science faculty in tertiary-level teaching hospitals who are not involved in patient care.
2. Participants who refused to provide informed consent.

### Data collection

We trained research physicians and nurses to collect data. We prepared the interviewing physicians and nurses regarding the project aim and objectives, ethics, outcome measurements, and comprehensive questionnaire. The study physicians and study nurses briefed each participant about the purpose of the study, risks and benefits, the anonymity of their identity and voluntary participations at the beginning of the face to face interview. Each participant enrolled has given written consent, permitting data usage for research. Our interviewers scheduled an appointment before the interview, and the face to face interview procedure strictly maintained all the COVID-19 safety protocols and maintained utmost hygiene. At enrolment, we collected their sociodemographic information such as age, gender, marital status, education, residing area, and occupation. We also collected information related to past illness or pre-existing co-morbid chronic conditions. We collected information about their development of symptoms and episodes of symptoms since the start of the COVID-19 pandemic. We asked the participants about their rt-PCR results or any other confirmatory tests performed to confirm SARS-CoV-2. We recorded the test results, whether it was positive or negative by rt-PCR, Serology, or other tests. We also inquired the participants about the usage of personal protective equipment (PPE) and the clinical settings where they could possibly encounter pathogens and availability during caregiving. We also asked if they treated either suspected or confirmed COVID-19 patients and the type of PPE they used with frequency. We collected information on where the HCWs attended the patients, such as indoors, in outpatient departments, in emergencies, or ICUs.

### Data management

We adapted the questionnaire into a smartphone application directly connected to a central server. The collected data are stored anonymously in the server as a spreadsheet document.

### Outcome

1. Lab confirmed COVID-19 among HCWs.

### Operational definition

Healthcare workers: “A healthcare worker was defined as all staff in the healthcare facility involved in the provision of care to patients, including those who may not have provided direct care to the patient but who may have had contact with the patient's body fluids, potentially contaminated items or environmental surfaces. This includes healthcare professionals, allied health workers, auxiliary health workers such as cleaning and laundry personnel, radiology physicians and technicians, clerks, phlebotomists, respiratory therapists, nutritionists, social workers, physical therapists, laboratory personnel, and cleaners, patient transporters, catering staff, etc. excluded HCWs of basic medical science faculty or administrative staff who are not involved in providing clinical care [15].

### Statistical analysis

We analyzed the outcomes by comparing them across various age groups, sexes, and other demographic categories, as well as clinical factors, SARS-CoV-2 exposure, and infection prevention and control (IPC) practices, including PPE usage, between HCWs with and without lab-confirmed COVID-19. Baseline characteristics of the HCWs were described using frequencies and percentages.

We conducted a bivariate analysis to compare data between HCWs with and without lab-confirmed COVID-19. Associations between individual factors or variables and positive or negative lab results were expressed as crude odds ratios (OR) in the bivariate analysis. Non-parametric tests, such as the chi-square test or Fisher's exact test, were used to assess the statistical significance of these associations. We also built a multivariable logistic regression model to adjust potential confounding variables to determine the variables independently associated with lab-confirmed COVID-19. We stratified the sample according to their sociodemographic, risk exposure in healthcare settings and community exposures, and IPC practices in different levels of wards and caregiving departments. In the adjusted model we considered the community exposure and IPC practice adherence as confounders along with comorbidities and role in the hospital settings and adjusted for the analysis. We also assessed the degree of association of the independent variables with the study outcome variable by adjusted odds ratio. The *p* value < 0.05 is also used as the level of significance.

## Results

### Characteristics of enrolled participants

We enrolled 3436 HCWs in the study. Among the participants, 26% (889) reportedly had SARS-CoV-2 by rt-PCR. The mean age of the participants was 38.1 years (SD 9.7 years) and 67% (2292) were female. Nearly half of the enrolled participants were nurses 47% (1632), followed by support staff 31% (1057), and physicians 22% (747). Among participants who tested positive for SARS-CoV-2, nurses made up 53%, followed by physicians at 30%, and support staff at 17%. The highest infection rates were seen in tertiary-level health facilities, with 71% (627) of HCWs. In addition, 36% (1234) of participants reported having other comorbid conditions, most commonly hypertension (32%) and chronic obstructive pulmonary disease (COPD) (19%). Of the SARS-CoV-2 positive cases, 78% reported exposure to confirmed COVID-19 patients through direct physical contact, or contact with body fluids, aerosols, or surfaces. Participants with positive PCR results also showed a higher prevalence of overweight, with 53% (473) having an elevated body mass index (BMI). At baseline, COVID-19 positive HCWs reported a range of symptoms: 81% (717) experienced fever, 55% (493) had a cough, 30% (267) reported a runny nose, 27% (239) had a sore throat, and 19% (169) experienced difficulty breathing. All participants experienced at least one or more symptoms associated with SARS-CoV-2 infection. In addition, 96% (668) of COVID-19 PCR-positive HCWs who were exposed during caregiving reported using proper PPE during patient care. However, the exposure to PPE usage was different in different facilities based on the healthcare tier and rural or urban settings (Table 1).

**Table 1:** Characteristics of enrolled HCWs

	Sampled health-care worker	Lab-confirm COVID-19	*p* value
	% (*n*)	% (*n*)	
Overall (*N* = 3436)	3436	26 (889)	
Age in year			
Mean ± SD	38.1 ± 9.7	38.7 ± 9.1	
< 25	5.97 (205)	1.8 (12)	
25–34	40.75 (1400)	38.5 (250)	< 0.001
35–44	28.41 (976)	31.4 (204)	
45–54	18.74 (644)	23.0 (149)	
≥ 55	6.14 (211)	5.2 (35)	
Sex			
Female	67 (2292)	69 (610)	0.222
Male	33.04 (1131)	31.38 (279)	
Type of HCW			
Physician	22 (747)	30 (266)	
Nurse	47 (1632)	53 (473)	< 0.001
Support Staff	31 (1057)	17 (150)	
Having a history of any co-morbid condition (*n* = 1234)		36% (1234)	
Cancer	0.8 (14)	1.06 (6)	0.769
Hypertension	32.42 (569)	31.33 (177)	0.598
Diabetes	21.88 (384)	23.19 (131)	0.908
HIV	0.11 (2)	0.18 (1)	0.524
Heart disease	5.58 (98)	5.84 (33)	0.909
Stroke	1.2 (21)	0.35 (2)	0.056
High blood cholesterol	8.83 (155)	9.91 (56)	0.467
Asthma	16.30 (286)	18.94 (107)	0.761
Chronic lung disease	1.31 (23)	1.95 (11)	0.116
Ever exposed or in contact with a confirmed COVID-19 patient	69.82 (2399)	78.18 (695)	< 0.001
Involved in direct COVID-19 patient care	94.96 (2278)	95.97 (667)	0.306
Wearing PPE	94.96 (2278)	96.12 (668)	0.248
Body-mass index (kg/m^2^)			
Mean ± SD	25.16 ± 8.5	26.10 ± 15.07	
Under-Weight	3.41 (117)	2.02 (18)	
Normal	47.85 (1644)	43.87 (390)	0.019
Over-Weight	47.70 (1639)	53.21 (473)	
Missing	1.4 (33)	1.4 (9)	
Symptoms experienced during COVID-19 illness			
Fever	20.90 (718)	81 (717)	
Cough	14.38 (494)	55 (493)	
Runny nose	7.77 (267)	30 (267)	< 0.001
Sore throat	6.98 (240)	27 (239)	
Difficulty in breathing	4.95 (170)	19 (169)	
Hospitalization due to COVID-19 complication	32 (356)	34 (354)	< 0.001
Type of healthcare facilities			
Tertiary	64.03 (2200)	70.53 (627)	
Secondary	14.76 (507)	15.07 (134)	< 0.001
Primary	21.22 (729)	14.40 (128)	

Heart disease: Refers to several types of heart conditions. The most common type of heart disease is coronary artery disease which affects the blood flow to the heart leading to heart failure

Cholesterol level: Blood cholesterol is fat substance essential for good health. High total cholesterol is 240 mg/dL or more. High cholesterol if untreated may lead to heart failure

Lung disease: Lung disease refers to several types of diseases or disorders that prevent the lungs from functioning properly. Lung disease can affect respiratory function, or the ability to breathe. E.g., Asthma, COPD, lung Cancer etc

Diabetes**:** Diabetes is a chronic disease that occurs either when the pancreas does not produce enough insulin or when the body cannot effectively use the insulin it produces. Insulin is a hormone that regulates blood glucose. If uncontrolled, it may cause detrimental health consequences

### Factors associated with COVID-19 infections

Compared with support staff, physicians and nurses were found to have a significantly higher risk of testing positive for COVID-19 after multivariable adjustment, with adjusted odds ratios (aORs) of 3.08 (95% CI 2.42–3.93) and 2.34 (95% CI 1.88–2.91), respectively. This association indicates that participants from primary-level facilities had a higher risk of COVID-19 compared to those in secondary-level facilities (aOR 1.55, 95% CI 1.16–2.08). Similarly, participants aged 35–44 years exhibited a slightly higher risk of contracting COVID-19 compared to other age groups (aOR 3.78, 95% CI 2.28–6.26). HCWs involved in direct COVID-19 patient care had a twofold higher risk of COVID-19 compared to those without such exposure (aOR 1.93, 95% CI 1.50–2.47; *p* < 0.001) (Table 2).

**Table 2:** Factors associated with SARS-COV-2 infections among HCWs, Bangladesh

	Unadjusted model	*p* value	Adjusted model	*p* value
	OR (95% CI)		OR (95% CI)	
Age in year				
25–34	3.1 (1.90–5.04)	0.001	2.4 (1.46–3.94)	0.001
35–44	4.4 (2.70–7.20)	< 0.001	3.78 (2.28–6.26)	< 0.001
45–54	3.89 (2.35–6.42)	< 0.001	3.36 (2.01–5.61)	< 0.001
≥ 55	2.8 (1.58–4.97)	< 0.001	3.18 (1.76–5.74)	< 0.001
< 25	Reference		Reference	
Type of HCW				
Physicians	3.34 (2.66–4.20)	< 0.001	3.08 (2.42–3.93)	< 0.001
Nurse	2.47 (2.01–3.02)	< 0.001	2.34 (1.88–2.91)	< 0.001
Support Staff	Reference		Reference	
Ever exposed or in contact with a confirmed COVID-19 patient				
Yes	2.18 (1.72–2.77)	< 0.001	1.93 (1.50–2.47)	0.001
No	Reference		Reference	
Type of healthcare facilities				
Tertiary	1.87 (1.51–2.31)	< 0.001	1.34 (1.06–1.70)	0.013
Secondary	1.67 (1.28–2.22)	< 0.001	1.55 (1.16–2.08)	0.003
Primary	Reference		Reference	

## Discussion

In this multicenter, hospital-based study with a substantial sample size, we present a detailed evaluation of the occupational risk of SARS-CoV-2 infection among HCWs across a range of healthcare facilities in Bangladesh. Our findings reveal that approximately a quarter of the HCWs surveyed (26%) were infected with COVID-19 during the pandemic. A systematic review by Dzinamaria et al. showed a pooled prevalence of COVID-19 was 11% ranging from 7 to 16% among the HCWs by PCR method. The prevalence is more than twofold higher among Bangladeshi HCWs [16]. This high prevalence underscores the significant vulnerability of this group, particularly in low- and middle-income countries (LMICs) where healthcare systems are often under-resourced and overburdened.

This study highlights multiple independent risk factors that are significantly linked to SARS-CoV-2 infection among HCWs. Specifically, Physicians and nurses were significantly more likely to contract COVID-19 than support staff, with adjusted odds ratios of 3.08 (95% CI 2.42–3.93) and 2.34 (95% CI 1.88–2.91), respectively. This finding aligns with other studies, including those from Malaysia, which similarly reported higher infection rates among female HCWs and nurses [17]. The elevated risk among these groups may be due to their extended close contact with patients, especially during emergencies or routine care, which increases their likelihood of virus exposure.

Our analysis also showed that HCWs in primary-level facilities had a higher risk of infection compared to those in secondary-level facilities (aOR 1.55, 95% CI 1.16–2.08). Relevant statistics regarding the risk of COVID-19 in different tiers of health facilities located in urban and rural areas in Bangladesh are scarce. However, studies showed that community people in rural areas are more prone to contracting COVID-19 than in urban areas which aligns with this study where all the primary health facilities lie in rural areas showing higher risk of COVID-19 among HCWs [18]. This finding highlights the challenges faced by lower-tier facilities, which often lack the necessary infrastructure and resources to implement effective infection prevention and control (IPC) measures. Moreover, primary-level facilities have fewer HCWs as a result despite contracting COVID-19 they had to attend to other patients increasing the risk of nosocomial spreading of the disease.

This study also found that direct exposure to COVID-19 patients was a significant risk factor, with 78% of lab-confirmed HCWs reporting exposure through direct contact, body fluids, or aerosol-generating procedures. Other studies suggest a similar probability of contracting COVID-19 with occupational exposure during caregiving [11, 19, 20]. In contrast, a study by Breeher et al. provides evidence that close contact during the aerosol-generating procedure is not associated with HCWs testing positive for SARS-CoV-2 [21] in line with the findings of another study by Vargese et al. where they found no association between any exposure-related factors and a positive COVID-19 case [22]. This suggests that, despite the use of PPE, the risk of infection remains high due to the nature of the work and potential lapses in PPE use or IPC practices.

Our findings also indicate that older age is a significant risk factor for infection (aOR 3.78, 95% CI 2.28–6.26), which is consistent with broader research identifying advancing age as a major risk factor for COVID-19 morbidity and mortality. This result is consistent with earlier research that links aging to an increased risk of COVID-19 illness [23–25]. This might be because frontline positions, which need regular and direct contact with infected patients, are more likely to be held by people in this age range. In addition, with advancing age, they become more susceptible to other co-morbid illnesses, which further exacerbates the severity of the current COVID-19 illness. In LMICs, where HCWs are frequently expected to work long into their senior years, this emphasizes the significance of routine screening and protective measures for older HCWs.

Interestingly, this study did not find a significant association between PPE usage and the reduction of lab-confirmed COVID-19 cases among HCWs. This finding contrasts with the findings of other studies where there is a drastic reduction of risk after usage of PPE in comparison to not using any PPE including face masks [3, 13, 19, 26]. However, this study did not investigate on reuse or improper use of PPE use. During the study, the HCWs did report on the scarcity of the supply of adequate PPE logistics. This raises concerns about PPE distribution, training, and compliance effectiveness in real-world settings, particularly in a healthcare system under significant strain.

This study also identified BMI of HCWs has a role in acquiring occupational risk of SARS-CoV-2 infection. We found that HCWs who were overweight showed higher chances of contracting COVID-19 in the bivariate model. However, even though the odds were higher in the multivariable model, the finding was not statistically significant. This result is similar to that of Malik et al., who discovered that a greater BMI was a significant risk factor for COVID-19 patients [27]. Male doctors with higher BMIs were linked to moderate and severe COVID-19, according to another study that also showed this fact for HCWs [28]. This finding could be resulting from condition where overweight increases oxidative stress resulting in limited breathing and, eventually, impairs the immune system's ability to fight off viral infections [29].

This study provides intriguing evidence of the occupational risks faced by HCWs in Bangladesh, offering a policy improvement for HCWs' safety in similar LMIC healthcare settings. The results emphasize how urgently IPC protocols need to be improved, especially in primary-level facilities where there is a greater risk of transmission. This includes not only the provision of adequate PPE but also ensuring proper training and adherence to IPC measures among all HCWs.

Strengthening the overall healthcare infrastructure, including better resource allocation to lower-tier facilities, is also critical. This will help ensure that all levels of healthcare facilities are equipped to manage infectious diseases effectively, thereby reducing the occupational risk for HCWs. The lessons learned from the COVID-19 pandemic should inform future strategies to bolster healthcare systems against potential epidemics.

## Limitations

This study has several limitations. First, we relied on self-reported information for laboratory confirmation of SARS-CoV-2, which, although verified where possible, may not be as reliable as direct specimen testing conducted during the study period. However, the reported data may create recall bias for the participants who had multiple episodes of illness and positive test results and may vary from the findings of similar studies with specimen testing. Second, we categorized HCWs based on their role in patient care, which may not fully capture the degree of exposure depending on the healthcare setting. Finally, the level of adherence to infection prevention and control (IPC) measures were based on self-reports, and the study did not include observational tools to verify actual practices in both COVID-19 and non-COVID-19 wards, which may also create reporting bias and depict an altered IPC practice to the interviewer. This mentioned bias may generate data that may differ from the findings from other studies in similar facilities where proper observational tools are implied.

## Recommendation

Our findings clearly underscore the necessity of implementing the prevention method of occupational exposure for HCWs through policy development of proper infection prevention and control measures protocol, comprehensive workshops and training, prioritizing an uninterrupted PPE supply chain management irrespective of COVID-19 and non-COVID-19 specialized hospitals.

## Conclusion

This study highlights the significant occupational risk of SARS-CoV-2 infection among HCWs in Bangladesh, particularly in primary-level facilities and among those in direct patient care roles. The findings will inform the policymakers and underscore the urgent need for targeted interventions such as adequate PPE supply to priority patient care settings, routine training on PPE usage or hygiene practices, and ensuring paid leave for infected staff to reduce nosocomial spread of the disease to enhance occupational safety, improve infection control, and strengthen healthcare systems, particularly in LMICs. Similar to the Bangladeshi healthcare system most of the LMICs have a scarcity of human resources and logistical support. This study provides insight into specific areas to strengthen to safeguard existing resources and ensure maximum benefit for HCWs and patients. As we prepare for future epidemics, we must draw on these insights to develop strategies that protect HCWs and ensure the sustainability of healthcare systems in the face of global health threats.

## Data Availability

The data set will be made available upon request to the corresponding author mentioning the reasonable purpose.
